# *Tinea capitis* in Older Adults: A Neglected and Misdiagnosed Scalp Infection—A Systematic Review of Reported Cases

**DOI:** 10.3390/antibiotics14121211

**Published:** 2025-12-01

**Authors:** Alfredo Valdez-Martinez, Mónica Ingrid Santoyo-Alejandre, Roberto Arenas, Mariel A. Isa-Pimentel, Juan Castillo-Cruz, Karla Daniela Huerta-Domínguez, Erika Fernanda Soto-Torres, Erick Martínez-Herrera, Rodolfo Pinto-Almazán

**Affiliations:** 1 Sección de Micología, Hospital General “Dr. Manuel Gea González”, Mexico City 14080, Mexico; alfredovaldezmart@gmail.com (A.V.-M.); rarenas98@hotmail.com (R.A.); 2 Facultad de Medicina, Universidad Nacional Autónoma de México (UNAM), Ciudad Universitaria, Mexico City 04510, Mexico; ingrid.santoyo.med@gmail.com (M.I.S.-A.); erikast.2022@gmail.com (E.F.S.-T.); 3 Co Coordinación de Residencia, Instituto Dermatológico Dominicano y Cirugía de Piel “Dr. Huberto Bogaert Díaz”, Santo Domingo 10306, Dominican Republic; isa_mariel@yahoo.com; 4 Sección de Estudios de Posgrado e Investigación, Escuela Superior de Medicina, Instituto Politécnico Nacional, Plan de San Luis y Díaz Mirón, Mexico City 11340, Mexico; juancast0508@gmail.com; 5 Hospital General del Zona 33, Instituto Mexicano del Seguro Social, Nayarit 63735, Mexico; karla.799081@gmail.com; 6 Fundación Vithas, Grupo Hospitalario Vithas, 28043 Madrid, Spain; 7 Efficiency, Quality, and Costs in Health Services Research Group (EFISALUD), Galicia Sur Health Research Institute (IISGS), Servizo Galego de Saúde-Universidade de Vigo (UVIGO), 36213 Vigo, Spain

**Keywords:** *Tinea capitis*, elderly, *Microsporum*, *Trichophyton*, inflammatory tinea, kerion, dry tinea, dermatophyte infection, scalp mycoses

## Abstract

**Background/Objectives**: *Tinea capitis* is traditionally a childhood infection, yet recent reports describe its emergence among older adults. In this population, hormonal changes, comorbidities, and frequent corticosteroid use may modify clinical presentation and delay diagnosis. This systematic review aimed to consolidate current evidence on *Tinea capitis* in individuals aged 65 years or older, focusing on epidemiologic, clinical, and mycological characteristics as well as therapeutic outcomes. **Methods**: Following PRISMA 2020 guidelines, a comprehensive search was conducted in the PubMed, Scopus, and SciELO databases for studies published between 1978 and February 2025. Eligible articles included case reports, case series, and clinical studies involving patients ≥65 years with confirmed *Tinea capitis*. Two independent reviewers screened and extracted data on demographics, comorbidities, risk factors, clinical manifestations, diagnostic methods, etiologic agents, and treatment response. **Results**: Sixty-three studies comprising 91 patients from 19 countries were included. Most cases originated from Spain (n = 27) and the United States (n = 12). Females accounted for 90.1% of cases. The leading comorbidities were diabetes mellitus (37.5%) and hypertension (21.9%). Topical corticosteroid use (40.7%) and pet exposure (27.8%) were frequent risk factors. Misdiagnosis occurred in 37.4% of patients, commonly as seborrheic dermatitis or erosive pustular dermatosis. The inflammatory variant predominated (65.9%), with kerion reported in 42.9%. *Microsporum canis* was the predominant agent (26.9%, n = 24), while *Trichophyton rubrum* and *Trichophyton tonsurans* were equally frequent (both 19.1%, n = 17). Systemic antifungal therapy achieved clinical cure in 91.2% of cases. **Conclusions**: *Tinea capitis* in the elderly is an underrecognized and often misidentified scalp infection. Awareness of its variable presentation and systematic mycological assessment are crucial to ensure timely therapy and prevent scarring alopecia.

## 1. Introduction

*Tinea capitis* is a superficial dermatophyte infection characterized by fungal invasion of the scalp and hair follicles, most commonly caused by species belonging to the genera *Trichophyton*, *Microsporum*, and *Nannizzia* [1,2,3]. This condition predominantly affects the pediatric population due to physiological features such as alkaline scalp pH, immature sebaceous gland activity, and hair morphology conducive to fungal adherence and invasion. In contrast, *Tinea capitis* is uncommon in adults and exceptionally rare among elderly individuals [1,2,4]. Nevertheless, recent case reports and small clinical series have increasingly documented *Tinea capitis* in older adults [1,5], particularly in postmenopausal women, drawing attention to an underrecognized and emerging clinical pattern within this age group. Although adult cases account for a minor fraction of global *Tinea capitis* reports—2.9% in Mexico and 4.2% in Egypt—epidemiological data indicate a gradual increase in incidence among older adults [6,7,8].

Multiple factors may account for this emergence. Age-related physiological and immunological alterations—including reduced estrogen levels, diminished sebum production, and progressive immunosenescence—compromise the scalp’s natural barrier function [1,9]. Furthermore, elderly individuals frequently exhibit comorbidities such as diabetes mellitus or require long-term immunosuppressive therapy (e.g., corticosteroids), both of which enhance susceptibility to dermatophyte infections [5,9,10,11]. Environmental and social determinants may also facilitate transmission, including residence in long-term care facilities, cohabitation within multigenerational households, and close contact with infected children. In these contexts, infection can spread through direct contact or indirectly via contaminated fomites such as hairbrushes, hats, or shared bedding [12,13]. Despite these risks, *Tinea capitis* in older adults remains underdiagnosed, in part due to its often atypical or subtle presentation, which may mimic other scalp conditions [1,13,14,15].

One of the severe inflammatory variants is kerion Celsi, characterized by a robust immune response that leads to painful nodular lesions, follicular destruction, and, if untreated, permanent scarring alopecia. Prompt recognition and early systemic treatment are critical to prevent irreversible sequelae [16,17]. In elderly patients, clinical manifestations may be subtle, variable, or resemble other dermatoses such as erosive pustular dermatosis, seborrheic dermatitis, psoriasis, or folliculitis decalvans. These atypical forms, combined with reduced diagnostic suspicion in this population, frequently result in misdiagnosis or delayed treatment, thereby complicating therapeutic management [15,16,17].

Systemic antifungal therapy remains the cornerstone of treatment for *Tinea capitis* across all age groups. Commonly used agents include allylamines (e.g., terbinafine), azoles (e.g., itraconazole, fluconazole), and griseofulvin. In refractory or complicated cases, echinocandins have been evaluated experimentally but are not routinely recommended for superficial infections. In elderly patients, antifungal selection should be individualized, considering pharmacokinetic changes, polypharmacy, and the potential for drug–drug interactions or adverse events [1,4,11,18].

Despite an increasing number of published case reports and clinical communications, *Tinea capitis* in the elderly remains an underexplored and neglected topic in dermatologic research. The available literature primarily consists of isolated case reports and small series, with no large-scale synthesis of epidemiological trends, clinical characteristics, or therapeutic outcomes, suggesting that the condition remains underrecognized and frequently undertreated in clinical practice [1].

**Figure 1:**
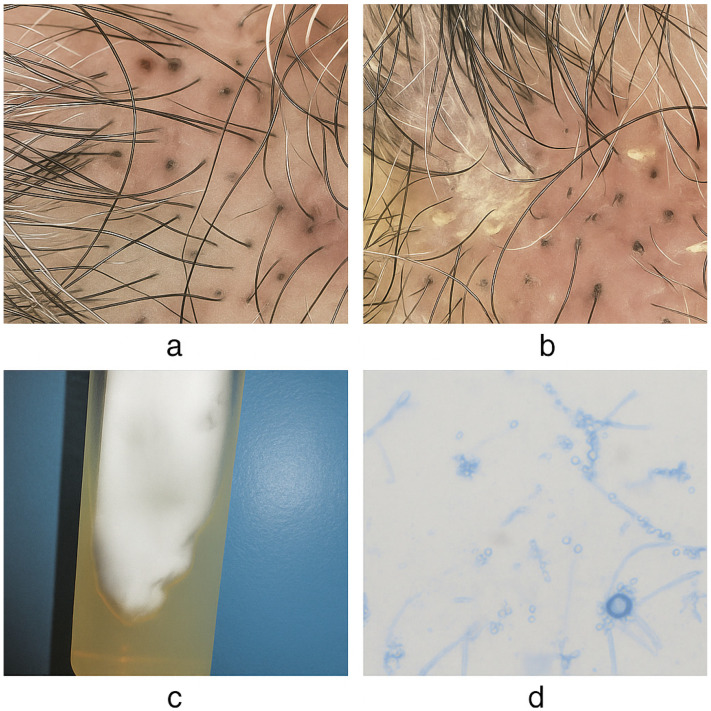
(**a**,**b**) Trichoscopy showing typical signs of *Tinea capitis*: “black dots” from hairs broken at the scalp surface, “comma hairs,” white perifollicular scaling, and hairs broken at varying lengths. (**c**) Culture on Sabouraud agar with velvety to powdery colonies and a whitish-cream coloration, consistent with *Trichophyton tonsurans*. (**d**) Microscopic image of *Trichophyton tonsurans* (lactophenol cotton blue, 400×): abundant sessile microconidia, some on short pedicels, distributed along the hyphae, with pyriform to rounded shapes.

This systematic review was designed in accordance with the PRISMA 2020 [19] statement to synthesize current evidence on *Tinea capitis* in individuals aged ≥65 years. The objectives were to describe epidemiological patterns, clinical and mycological features, predisposing factors, diagnostic modalities, and therapeutic approaches reported in this population. By elucidating the distinctive aspects of *Tinea capitis* in older adults, this review aims to enhance clinical recognition, inform evidence-based management, and identify knowledge gaps warranting further investigation (Figure 1).

## 2. Results

A total of 63 studies were included, comprising 91 patients aged ≥65 years diagnosed with *Tinea capitis*. The included studies spanned from 1978 to 2025, with data drawn from 19 countries (Table 1) [5,16,20,21,22,23,24,25,26,27,28,29,30,31,32,33,34,35,36,37,38,39,40,41,42,43,44,45,46,47,48,49,50,51,52,53,54,55,56,57,58,59,60,61,62,63,64,65,66,67,68,69,70,71,72,73,74,75,76,77,78,79,80].

### 2.1. Demographic and Geographic Characteristics

**Figure 2:**
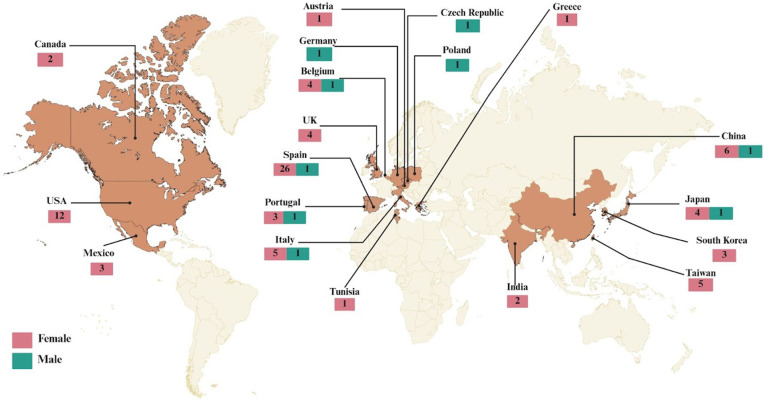
Geographic Distribution of *Tinea capitis* in elderly patients.

The majority of reported cases were from Spain (n = 27), with a notable distribution also in the United States (n = 12), China (n = 7) and Italy (n = 6). Regarding sex distribution, 90.1% of cases (n = 82) occurred in females, while 9.9% (n = 9) involved males. All patients included were aged ≥65 years; most cases occurred in individuals aged 65–74 years (n = 44) followed by those aged 75–84 years (n = 31) and patients aged 85 years or older (n = 16) (Figure 2).

### 2.2. Comorbidities and Risk Factors

Comorbidities were reported in 30 patients. The most prevalent were diabetes mellitus (37.5%, n = 12), hypertension (21.9%, n = 7), Other commonly reported included cancer (18.8%, n = 6), autoimmune diseases (15.6%, n = 5), actinic keratosis (12.5%, n = 4), and malnutrition (9.4%, n = 3). In addition, a variety of less frequent comorbidities were documented in 31.3% of patients (n = 10).

Regarding risk factors, 59.4% of patients (n = 54) presented with at least one identifiable risk factor. The use of topical glucocorticoids emerged as the most frequent factor (40.7%, n = 22), followed by pet exposure (27.8%, n = 15)—most commonly to cats and dogs and, less frequently, to small mammals such as hamsters and rabbits—and systemic glucocorticoid use (16.7%, n = 9). Furthermore, both residence in a nursing home and treatment with immunosuppressive agents were reported in 13.0% of patients (n = 7, respectively). Notably, a family history of *Tinea* and recent attendance at hair salons were each reported in 11.1% of cases (n = 6).

Coexisting dermatophytosis were documented in 27.5% of patients (n = 25); the most common were *Tinea faciei* and *Tinea unguium* (n = 11, each), followed by *Tinea corporis* and *Tinea pedis* (n = 7, each). In 12 cases, more than one concurrent dermatophytosis was identified.

A further relevant finding was that 37.4% of cases (n = 34) were initially misdiagnosed. The most frequent misdiagnoses were seborrheic dermatitis (n = 9) and erosive pustular dermatosis (n = 8).

### 2.3. Clinical Presentation

The mean time from symptom onset to diagnosis was 477.3 days. Lesions most commonly involved the parietal (62.6%) and occipital (24.2%) regions, with less frequent involvement of the frontal and temporal areas (each reported in 19.8% of cases). The most frequently observed primary lesions were pustules (n = 59) and papules (n = 37). Among secondary lesions, scaling was present in 66 cases, crusting in 59, and ulceration in 11. The most commonly reported associated symptoms were pruritus (n = 38) and pain (n = 22). Erythema was noted in 97.8% of cases (n = 89), and alopecia was observed in all reported cases (n = 91).

Clinically, the most common presentation was the inflammatory *Tinea capitis* (n = 43); kerion was reported in 90.6% (n = 39), while favus variant was identified in 9.3% (n = 4). The non-inflammatory *Tinea capitis* was observed in 34.1% of cases (n = 31). Detailed clinical features, comorbidities, and outcomes are provided in Appendix A (Table A1).

### 2.4. Mycological Findings

Diagnosis was established through direct microscopic examination, culture, and, in selected cases, histopathology and polymerase chain reaction (PCR). Direct examination was performed in 64 cases, with positive findings reported in 82.8% (n = 53). Cultures were conducted in 90 cases and yielded positive results in 97.8% (n = 88). Histopathological examination was carried out in 34 patients, confirming the diagnosis in 70.6% (n = 24). Wood’s lamp examination was performed in 19 cases, with fluorescence observed in 47.4% (n = 9). PCR testing was employed in 9 cases, all of which returned positive results (100%). Additionally, dermoscopy was performed in 16.5% of cases (n = 15). The most frequent dermoscopic features were broken hairs (n = 8, 53.3%) and comma hairs (n = 7, 46.7%). Less common findings included black dots, zigzag hairs, bar-code-like hairs, perifollicular scaling and erythema.

Etiological agents were identified in 89 of the 91 cases. The most commonly isolated species were *Microsporum canis* (26.9%, n = 24), *Trichophyton tonsurans* (19.1%, n = 17), *Trichophyton rubrum* (19.1%, n = 17), and *Trichophyton violaceum* (16.9%, n = 15). Less frequently reported organisms included *Nannizzia gypsea* (4.5%, n = 4), *Trichophyton schoenleinii* and *Microsporum* spp. (3.4%, n = 3 each), followed by *Microsporum audouinii*, *Trichophyton mentagrophytes*, and *Trichophyton* spp. (2.2%, n = 2 each).

### 2.5. Treatment and Management

Systemic antifungal treatment was administered in 93.4% of patients (n = 85). In contrast, 3.3% (n = 3) did not receive treatment, and in another 3.3% (n = 3), treatment status was not reported.

The most frequently used drug class was allylamines, prescribed in 52.9% of treated cases (n = 45), followed by benzofurans (22.4%, n = 19) and azoles (17.6%, n = 15). Less commonly used agents included echinocandins (8.2%, n = 7) and systemic corticosteroids (5.9%, n = 5).

Topical antifungal treatment was administered in 30.8% of patients (n = 28), while 66% (n = 60) did not receive topical therapy. In 3.3% of cases (n = 3), treatment information was not reported.

Among those treated topically, the most commonly used drug types were azoles (60.7%, n = 17), followed by ointments of unspecified class (14.3%, n = 4), allylamines (14.3%, n = 4), corticosteroids (10.7%, n = 3), hydroxypyridones (10.7%, n = 3), and morpholines (3.6%, n = 1).

A total of 28.6% of patients (n = 26) received both systemic and topical antifungal therapy.

Clinical resolution was achieved in 97.6% (n = 82) of the 85 patients who received systemic antifungal therapy. Among those treated topically (n = 28), 89.3% (n = 25) achieved resolution of lesions.

Overall, 91.2% of patients (n = 83) experienced complete clinical recovery, 3.3% (n = 3) did not achieve clinical improvement, and outcomes were not reported in 5.5% of cases (n = 5).

### 2.6. Statistical Analyses

The odds ratios for the associations between cure and sex, risk factors, comorbidities, etiological agent, *Tinea* type (inflammatory or non-inflammatory) as well as treatment are presented in Table 2, determined through univariate and multivariate analyses.

In the univariate analysis, those who received treatment were substantially more likely to achieve clinical cure compared to untreated patients (OR = 136.7, 95% CI [14.88–1597]; *p* < 0.0001). Additionally, the presence of risk factors showed a tendency toward a higher probability of cure (OR = 4.324, 95% CI [0.9890–21.73]; *p* = 0.064); however, this association did not reach statistical significance.

In the multivariate logistic regression model, treatment remained an independent predictor of cure (OR = 324.3, 95% CI [18.88–21,695]; *p* = 0.0008), indicating a markedly higher likelihood of recovery among treated individuals.

A logistic regression analysis was performed to predict the probability of success. The model intercept (OR = 0.009206, 95% CI [0.0001319–0.1221]; *p* = 0.005) indicated that the odds of success were significantly lower in the absence of other predictive factors.

## 3. Discussion

*Tinea capitis* in older adults remains an uncommon but increasingly recognized clinical entity [1,81,82]. In this review, we identified 63 studies reporting 91 patients aged ≥65 years from 19 countries between 1978 and 2025 [5,16,20,21,22,23,24,25,26,27,28,29,30,31,32,33,34,35,36,37,38,39,40,41,42,43,44,45,46,47,48,49,50,51,52,53,54,55,56,57,58,59,60,61,62,63,64,65,66,67,68,69,70,71,72,73,74,75,76,77,78,79,80]. The predominance of reports from Spain (n = 27), followed by the United States (n = 12), China (n = 7), and Italy (n = 6), may reflect both genuine epidemiologic patterns and regional publication activity rather than true differences in incidence. Comparable geographic trends have been documented in adult dermatophytosis, where Mediterranean countries consistently report higher diagnostic awareness and laboratory confirmation rates [81,83]. Notably, in our systematic review we did not identify any cases from South America and only very few reports from other low- and middle-income regions, despite the broad time frame of our search. This geographic gap most likely reflects underreporting rather than a true absence of disease, driven by factors such as limited access to specialized mycological diagnostics, competing clinical and research priorities, and a lower likelihood of publishing single cases or small series in indexed journals. In addition, variable awareness of *Tinea capitis* in older adults among clinicians may lead to misclassification as other scalp dermatoses, especially in settings where routine mycological examination is not performed. These aspects highlight the need to strengthen surveillance and reporting of superficial mycoses in older adults, particularly in underrepresented regions.

A striking finding was the marked female predominance (90.1%), which inverses the pediatric pattern [4,82]. This difference likely stems from a complex interplay of hormonal, biological, and behavioral factors. In postmenopausal women, estrogen deficiency alters scalp physiology by reducing sebaceous gland activity, lipid composition, and epidermal barrier function, thereby diminishing the natural fungistatic defense of the scalp [84,85]. Additionally, cosmetic hair practices and increased healthcare-seeking behavior among elderly women may favor detection and transmission [22,73], whereas androgenetic alopecia in elderly men reduces terminal hair follicles, limiting fungal colonization [86].

The age distribution—most cases in the 65–74-year group (n = 44)—underscores that the infection may occur early in senescence and persist through advanced age [1,63,82]. Over the decades analyzed, the persistence of *M. canis* as the predominant agent suggests continued zoonotic exposure in urbanized populations, likely linked to pet ownership, despite the expected shift toward anthropophilic species such as *T. tonsurans* [25,82,87].

Comorbidities were reported in 30 patients, most frequently diabetes mellitus (37.5%) and hypertension (21.9%). These reflect not only the general geriatric background but also their pathophysiological contribution to susceptibility [88,89,90]. Diabetes impairs neutrophil chemotaxis and local vascular perfusion, while hypertension correlates with endothelial dysfunction, both of which reduce cutaneous immune response [88,89,90]. Malnutrition and malignancy, present in nearly one-third of cases, further exacerbate immunosenescence [91,92].

Topical corticosteroid use was the predominant risk factor (40.7%), emphasizing its iatrogenic potential in transforming classic fungal lesions into tinea incognito by suppressing local immunity and altering morphology [20]. Pet exposure (27.8%) remains a major zoonotic route for *M. canis* [25], whereas systemic corticosteroids, nursing home residence, and immunosuppressive therapy (each 13%) reflect both pharmacological and institutional vulnerability [1,13,22]. Coexistent dermatophytoses were observed in 27.5%, primarily *T. unguium* and *T. faciei*, which may serve as reservoirs for scalp reinfection [17,25].

A critical finding is that 37.4% of cases were initially misdiagnosed, most often as seborrheic dermatitis or erosive pustular dermatosis. Such diagnostic errors prolong disease duration, contribute to scarring alopecia, and increase healthcare costs [1,3,16,36]. Increased clinical suspicion and early mycological testing are therefore essential, especially in patients with chronic or steroid-modified scalp lesions [1,20].

The mean diagnostic delay of 477 days highlights underrecognition of *Tinea capitis* in older adults. Aging skin exhibits decreased Langerhans cell density, impaired keratinocyte signaling, and diminished sebaceous lipids, collectively predisposing to persistent colonization and delayed inflammatory recognition [91,92,93,94]. Lesions most commonly affected the parietal (62.6%) and occipital (24.2%) regions, and alopecia was universal [1,17].

Despite the expected immune decline with aging, inflammatory variants predominated (n = 43). Kerion was documented in 90.6% of these cases, suggesting that elderly immune systems can still mount robust inflammatory responses to dermatophyte antigens [1,17,26,31]. The persistence of favus (n = 4) underlines chronicity and potential neglect. These findings emphasize that both intense inflammation and chronic non-inflammatory forms may coexist in this population [27,39].

Direct microscopy (82.8% positive) and culture (97.8% positive) remained the reference diagnostic methods in our cohort, consistent with contemporary reviews that position KOH/culture as the gold standard for *Tinea capitis* in adults [1,17]. Histopathology contributed diagnostic value (70.6% positive in our series), particularly as a supportive tool in inflammatory presentations; however, in kerion the intense suppurative response can fragment or eliminate fungal elements, and both histology and mycological studies may be falsely negative. Accordingly, the absence of visible hyphae on biopsy (even with PAS/GMS) does not exclude *Tinea capitis* when clinical suspicion is high [95,96,97]. In such scenarios, diagnostic yield improves by sampling the active peripheral margin, epilating hairs from less inflamed areas or from concomitant non-inflammatory plaques, repeating collection after local care, and, where available, incorporating fungal PCR/ITS assays [17,96,98].

Dermoscopy—reported in 16.5% of cases—has gained prominence as a non-invasive adjunct for both diagnosis and follow-up. Characteristic patterns (comma, corkscrew, zigzag, Morse-code hairs) show robust diagnostic performance and can guide targeted sampling and monitor treatment response, helping to reduce the need for biopsy in older adults [17,56,68,69,99].

The etiological spectrum was heterogeneous. *M. canis* was most frequent (26.9%), followed by *T. tonsurans* (19.1%), *T. rubrum* (19.1%), and *T. violaceum* (16.9%). The concurrent presence of zoophilic, anthropophilic, and geophilic agents—including *N. gypsea* (4.5%)—supports multiple transmission routes in geriatric populations (pet contact, healthcare environments, and soil exposure) [1,42,59].

Systemic antifungal therapy was administered in 93.4% of patients, confirming its central therapeutic role. Allylamines were most frequently used (52.9%), followed by benzofurans (22.4%) and azoles (17.6%). Clinical resolution was achieved in 97.6% of those receiving systemic therapy and in 89.3% of those treated topically, yielding an overall recovery rate of 91.2%. The high efficacy observed may reflect adequate drug absorption and shorter disease duration once diagnosed [1,17]. Notably, *M. canis* infections responded to terbinafine monotherapy in several cases, challenging earlier assumptions of reduced susceptibility and aligning with human pharmacokinetic studies showing high and persistent concentrations in the stratum corneum and sebum in adults [16,22,61,73,79,100]. Griseofulvin and azoles were typically used when terbinafine was unavailable or contraindicated, and a combined systemic–topical approach (28.6%) was employed to reduce fungal burden and transmissibility [17].

In our review, systemic antifungal therapy was administered in the large majority of patients, whereas only a small subset received topical antifungals alone. This pattern reflects the fact that, in older adults, scalp infection is usually extensive, chronic, or associated with comorbidities, favoring systemic regimens. Topical agents may be considered as monotherapy only in highly selected cases—such as small, superficial, non-scarring plaques in patients without significant immunosuppression and with good adherence—or as adjunctive therapy together with antiseptic shampoos to reduce fungal burden and transmissibility. Even in these scenarios, careful follow-up is recommended to ensure clinical and mycological cure and to prevent progression to scarring alopecia [17,101].

Agent selection should consider the likely species when available: terbinafine performs particularly well against *Trichophyton*, whereas classic data support griseofulvin when *Microsporum* is strongly suspected—without delaying treatment initiation [4,17,100]. In adults, reference regimens include terbinafine 250 mg/day for 4–6 weeks (often extended when *Microsporum* is suspected) and griseofulvin for 6–8+ weeks; shampoos (ketoconazole or selenium sulfide) are recommended as adjuncts 2–3 times weekly for the patient and close contacts to decrease spore load and relapse [17,101].

Although cure rates exceeded 90%, management in older adults should account for age-related pharmacokinetics, hepatic metabolism, and polypharmacy [102,103]. Baseline liver function testing is reasonable before initiating terbinafine, with clinical monitoring thereafter [7,10]. Drug–drug interactions merit particular attention: terbinafine inhibits CYP2D6 (with possible effects on certain antidepressants, β-blockers, and class IC antiarrhythmics), griseofulvin may interact with warfarin, and azoles (e.g., itraconazole) can prolong QT and inhibit CYP3A4 [100,102,103,104,105].

Echinocandins were rarely used (8.2%) and are not recommended as routine therapy for scalp dermatophytosis; evidence is limited despite favorable in vitro activity [101]. Systemic corticosteroids (5.9%) may be considered briefly as adjuncts in severe kerion to reduce edema, pain, and potential scarring, but only alongside systemic antifungals and with individualized risk–benefit assessment [15]. Treatment failures (3.3%) likely reflect antifungal resistance, poor adherence, or profound immunosuppression; when failure occurs despite adequate dosing and adherence, culture with susceptibility testing and/or switching class (e.g., to itraconazole) should be considered, keeping in mind the emergence of terbinafine-resistant *T. indotineae* [101,106].

Dermoscopy is useful for treatment monitoring, documenting the disappearance of comma/corkscrew/zigzag/Morse-code hairs and the emergence of regrowing hairs, which correlates with clinical improvement and can reduce repeated cultures [99,107]. Finally, reducing relapse requires addressing reservoirs: treat concurrent *tinea* (e.g., *unguium*, *faciei*), maintain adjunctive shampoos during the first weeks, and, in suspected *M. canis,* evaluate pets and environmental contamination to prevent reinfection [25,101].

## 4. Materials and Methods

**Figure 3:**
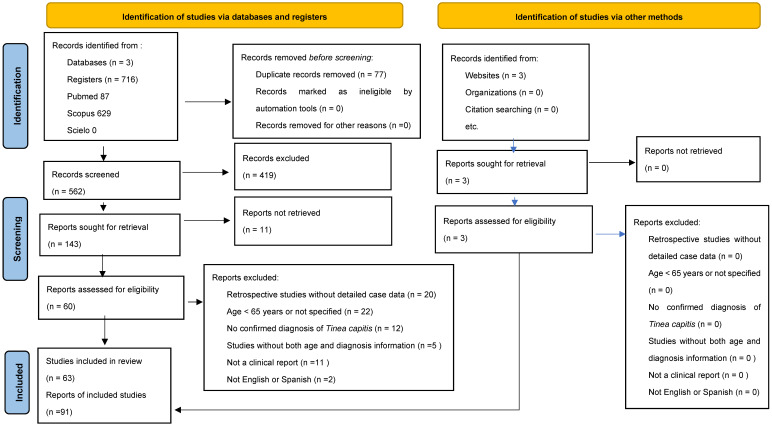
Prisma 2020 flowchart of the data extracted for the systematic review.

This systematic review was designed and executed in accordance with the PRISMA 2020 (Preferred Reporting Items for Systematic Reviews and Meta-Analyses, PROSPERO code: CRD420251001550) guidelines (Figure 3). An exhaustive literature search was carried out across three major databases—PubMed, Scopus, and SciELO—aimed at identifying publications classified under Adaptive Clinical Trials, Case Reports, Classical Articles, Clinical Studies, Comparative Studies, Editorials, Evaluation Studies, Letters, Multicenter Studies, Observational Studies, and Twin Studies. The search strategy employed the Medical Subject Headings (MeSH) terms “*Tinea capitis*” OR “Kerion Celsi.” In both PubMed and Scopus, search filters were applied to limit results to studies involving human subjects aged 65 years or older. Furthermore, only articles published in English or Spanish were considered eligible for inclusion. The temporal scope of included publications extended from 1978 to February 2025, thereby providing a comprehensive historical overview of the subject.

Two independent reviewers (Valdez and Santoyo) performed a rigorous screening process, assessing titles, abstracts, and full texts of all potentially relevant studies. Final inclusion decisions were made collaboratively, with discrepancies resolved through discussion with Arenas, Pinto-Almazán, and Martínez-Herrera, who jointly finalized the eligibility criteria. Selection of studies and data items: We removed duplicate records and included reports that provided a minimum core dataset—patient age, confirmed *Tinea capitis* diagnosis, the diagnostic method used, the etiologic agent, and whether treatment was administered (systemic and/or topical). Studies lacking any of these core items were excluded. After primary selection, we screened reference lists of all included articles; no additional studies were found via citation chasing. We also identified three additional reports through targeted searches of reputable websites (treated as gray literature) and included them only if they met the same eligibility criteria.

### 4.1. Quality Assessment

We appraised case quality using the Joanna Briggs Institute (JBI) Critical Appraisal Checklist for Case Reports. Our review covered eligibility criteria, patient history, clinical presentation, interventions, and outcomes. Two reviewers (Valdez-Martinez and Santoyo-Alejandre) independently judged the methodological quality of each report. Overall review quality was benchmarked against PRISMA 2020 guidelines. A meta-analysis was not undertaken due to insufficient quantitative data across the included case reports.

### 4.2. Statistical Evaluation

We conducted descriptive statistics to profile the study’s quantitative and qualitative variables. Chi-square tests were employed for univariate assessments of associations, adopting *p* ≤ 0.05 as the threshold for statistical significance. Clinical cure served as the outcome in a multivariable logistic regression to identify independent predictors. All computations were performed in GraphPad Prism 9.0 (GraphPad, San Diego, CA, USA).

## 5. Conclusions

This review shows that *T. capitis* in older adults is a multifactorial infection shaped by hormonal decline, immunosenescence, behavioral factors, and iatrogenic exposures. Its high misdiagnosis rate (37.4%) and prolonged diagnostic delay highlight the need for stronger awareness and earlier mycological testing among dermatologists and general practitioners. Strengthening dermoscopic skills, improving sampling strategies in inflammatory variants and addressing reservoirs (nails, pets, environment) are practical levers to reduce time-to-diagnosis and relapse.

## Figures and Tables

**Table 1:** Overview of published cases of *Tinea capitis* in elderly patients, including demographic data, comorbidities, etiological agents, diagnostic methods, and treatment.

Year	Country	Sex	Age	Risk Factors and Comorbidity	Topography	Another Dermatophytosis	Diagnostic Method			Agent	Treatment		Ref.
							Direct Exam	Culture	Histopathology		Oral	Topic	
2007	Austria	F	76	TCS	Occipital	*Tinea corporis*	+	+	NA	*M. canis*	Yes	Yes	[20]
1980	Belgium	F	75	CA/PE, FM	Occipital	No	+	+	+	*M. canis*	Yes	No	[21]
2014		F	72	TCS, NH, HS	Parietal	No	NA	+	+	*M. canis*	Yes	No	[22]
2014		M	90	HS	NA	No	+	+	NA	*M. canis*	Yes	No	[22]
2014		F	93	HS, NH	NA	No	+	+	NA	*M. canis*	Yes	No	[22]
2014		F	93	HS, NH	NA	No	+	+	NA	*M. canis*	Yes	No	[22]
1995	Canada	F	90	NH	Parietal, Temporal	*Tinea unguium*	+	-	NA	*T. rubrum*	Yes	No	[23]
2000		F	85	HTN, AK, HT	Parietal	No	NA	+	NA	*T. rubrum*	Yes	No	[24]
2021	China	F	71	DM/PE	Parietal	*Tinea unguium*, *Tinea corporis*, *Tinea cruris*, *Tinea faciei*, *Tinea pedis*	+	+	NA	*M. canis*	Yes	Yes	[25]
2022		F	75	Maln.	Parietal, Temporal	*Tinea unguium*, *Tinea pedis*	+	+	+	*T. rubrum*	Yes	No	[26]
2022		F	70	HT	Occipital, Frontal, Parietal, Temporal	*Tinea faciei*, *Tinea corporis*	+	+	+	*T. schoenleinii*	Yes	No	[27]
2023		F	68	PE	Occipital, Parietal, Frontal	No	+	+	NA	*T. mentagrophytes*	Yes	Yes	[28]
2023		M	77	None	Parietal	*Tinea pedis*, *Tinea unguium*	+	+	NA	*T. rubrum*	Yes	Yes	[29]
2024		F	66	OCS, HD	Parietal, Frontal	No	+	+	+	*T. rubrum*	Yes	Yes	[30]
2025		F	69	None	Occipital	No	+	+	+	*T. tonsurans*	Yes	Yes	[31]
2010	Czech Republic	M	83	None	Frontal, Parietal	*Tinea pedis*, *Tinea unguium*	+	+	+	*T. rubrum*	Yes	Yes	[32]
2005	Germany	M	65	None	Parietal	No	-	+	-	*T. rubrum*	Yes	Yes	[33]
2011	Greece	F	100	Gast., Maln.	Frontal, Parietal	No	+	+	NA	*M. canis*	Yes	No	[34]
2006	India	F	70	None	Occipital	No	+	+	+	*T. violaceum*	Yes	No	[35]
2014		F	70	None	Occipital, Parietal	No	+	+	NA	*T. rubrum*	Yes	No	[36]
1986	Italy	F	68	PE	Parietal, Frontal	*Tinea faciei*	+	+	NA	*M. canis*	Yes	No	[37]
1995		F	84	PE, FM	Parietal	No	+	+	NA	*M. canis*	Yes	Yes	[38]
2003		M	87	CA	Temporal	*Tinea faciei*	+	+	NA	*T. schoenleinii*	Yes	No	[39]
2019		F	79	AID/OCS, TCS, IS, PE	Parietal, Occipital	No	+	+	NA	*M. canis*	Yes	Yes	[40]
2022		F	66	None	Parietal, Temporal, Frontal	No	NA	+	+	*T. tonsurans*	Yes	Yes	[41]
1998		F	69	PE	Parietal	No	NA	+	NA	*N. gypsea*	Yes	No	[42]
1999	Japan	M	75	TCS	Temporal, Occipital	*Tinea cruris*, *Tinea pedis*, *Tinea unguium*	NA	+	-	*T. rubrum*	Yes	No	[43]
2004		F	85	LD/NH	Parietal	*Tinea cruris*	+	+	NA	*T. violaceum*	No	Yes	[44]
2011		F	66	FM	Parietal, Temporal	No	+	+	NA	*T. violaceum*	Yes	No	[45]
2016		F	66	PE, OCS	Occipital	No	NA	+	NA	*M. canis*	Yes	No	[46]
2021		F	82	TCS	Temporal	*Tinea corporis*, *Tinea unguium*	+	+	NA	*T. violaceum*	Yes	No	[47]
2002	Mexico	F	75	HTN	Parietal	*Tinea corporis*, *Tinea faciei*	+	+	NA	*T. tonsurans*	Yes	Yes	[48]
2005		F	67	DM	Parietal	*Tinea faciei*	+	+	NA	*T. tonsurans*	Yes	No	[49]
2015		F	68	FM	Parietal	No	+	+	NA	*T. tonsurans*	Yes	No	[50]
2024	Poland	M	75	None	Occipital	2 feet 1 hand syndrome, *Tinea unguium*, Tinea barbae	NA	+	NA	*T. rubrum*	Yes	Yes	[51]
2019	Portugal	F	92	Inf./TCS	Occipital, Parietal, Temporal, Frontal	No	NA	+	+	*M. audouinii*	No	No	[52]
2019		M	86	None	NA	No	+	+	NA	*T. rubrum*	Yes	No	[53]
2019		F	71	DM, D	NA	*Tinea pedis*, *Tinea unguium*	-	+	NA	*T. tonsurans*	Yes	No	[53]
2019		F	86	HIV	NA	*Tinea faciei*	+	+	NA	*T. violaceum*	Yes	No	[53]
2020	South Korea	F	82	DM/PE	Frontal, Parietal	*Tinea faciei*, *Tinea corporis*	+	+	+	*M. canis*	Yes	Yes	[54]
2022		F	90	CA, Inf.	Occipital, Parietal	No	+	NA	-	NA	No	Yes	[55]
2024		F	82	HTN/PE	Parietal	No	NA	+	NA	*M. canis*	Yes	Yes	[56]
2002	Spain	F	75	DM	Occipital, Parietal, Temporal, Frontal	No	-	+	-	*T. violaceum*	Yes	No	[57]
2002		F	70	HTN/TCS	Frontal, Parietal	No	+	+	+	*T. violaceum*	Yes	Yes	[58]
2004		F	70	TCS	Parietal	*Tinea corporis*	NA	+	+	*N. gypsea*	Yes	Yes	[59]
2004		M	77	CA/OCS, IS	Parietal, Temporal, Frontal	Tinea barbae	+	+	NA	*T. violaceum*	Yes	Yes	[60]
2006		F	76	HTN/PE, TCS	Occipital, Parietal	*Tinea faciei*	NA	+	NA	*M. canis*	Yes	No	[61]
2007		F	65	TCS	Parietal	No	+	+	NA	*T. violaceum*	Yes	Yes	[62]
2012		F	71	TCS	Parietal, Occipital	No	+	+	NA	*T. tonsurans*	Yes	No	[63]
2012		F	65	HTN DM	Parietal	No	NA	+	-	*T. rubrum*	Yes	No	[63]
2012		F	69	None	Occipital	No	NA	+	NA	*T. tonsurans*	Yes	No	[63]
2012		F	72	TCS	Parietal, Temporal, Frontal	No	NA	+	+	*T. mentagrophytes*	Yes	No	[63]
2015		F	84	PE, TCS	Temporal, Parietal, Frontal	No	NA	+	+	*N. gypsea*	Yes	No	[64]
2016		F	76	None	NA	No	-	+	NA	*T. violaceum*	Yes	No	[5]
2016		F	74	None	NA	No	-	+	NA	*T. violaceum*	Yes	No	[5]
2016		F	75	None	NA	No	-	+	NA	*T. violaceum*	Yes	No	[5]
2016		F	68	None	NA	No	+	+	NA	*M. canis*	Yes	No	[5]
2016		F	70	None	NA	No	+	+	NA	*M. canis*	Yes	No	[5]
2016		F	80	None	NA	No	+	+	NA	*N. gypsea*	Yes	No	[5]
2016		F	75	None	NA	No	-	+	NA	*T. violaceum*	Yes	No	[5]
2016		F	80	None	NA	No	+	+	NA	*M. canis*	Yes	No	[5]
2016		F	71	None	NA	No	+	+	NA	*T. tonsurans*	Yes	No	[5]
2016		F	74	OCS	NA	*Tinea faciei*	+	+	NA	*M. canis*	Yes	No	[5]
2016		F	75	OCS	NA	No	+	+	NA	*T. violaceum*	Yes	No	[5]
2016		F	70	IS	NA	No	+	+	NA	*M. canis*	Yes	No	[5]
2016		F	71	IS	NA	No	-	+	NA	*T. tonsurans*	Yes	No	[5]
2016		F	71	TCS	NA	No	-	+	NA	*T. tonsurans*	Yes	No	[5]
2021		F	78	None	Parietal, Temporal	*Tinea unguium*, *Tinea pedis*	+	+	-	*T. rubrum*	Yes	Yes	[65]
2021		F	73	AID	Occipital	No	NA	+	NA	*M. canis*	Yes	No	[66]
1991	Taiwan	F	66	FM	Parietal	No	+	+	NA	*T. violaceum*	Yes	No	[67]
1991		F	72	None	Parietal	No	+	+	NA	*T. rubrum*	Yes	No	[67]
1991		F	70	OCS	Parietal	No	+	+	NA	*T. rubrum*	Yes	No	[67]
2014		F	65	None	Parietal	No	+	-	NA	NA	NA	NA	[68]
2015		F	79	None	Occipital	*Tinea faciei*	NA	+	+	*M. audouinii*	NA	NA	[69]
2012	Tunisia	F	73	None	Parietal, Temporal, Frontal	*Tinea unguium*	+	+	NA	*T. schoenleinii*	Yes	Yes	[70]
1978	United Kingdom	F	76	PE	Frontal	No	NA	+	NA	*M. canis*	Yes	Yes	[71]
1994		F	83	None	Parietal	No	NA	+	-	*T. tonsurans*	Yes	Yes	[72]
2001		F	71	HS	Parietal, Occipital	No	NA	+	+	*M. canis*	Yes	No	[73]
2001		F	71	AID/IS, HS	Parietal	No	NA	+	+	*M. canis*	Yes	No	[73]
1980	USA	F	86	IHD/FM	Frontal, Occipital	No	+	+	NA	*T. tonsurans*	Yes	No	[74]
1993		F	67	AID/TCS	Parietal	No	+	+	NA	*T. rubrum*	Yes	Yes	[75]
2002		F	67	TCS	Parietal	No	+	+	NA	*T. tonsurans*	NA	NA	[76]
2003		F	87	PE, OCS	Occipital, Parietal, Temporal	No	NA	+	+	*T. tonsurans*	Yes	Yes	[77]
2003		F	75	TCS	Parietal	No	NA	+	-	*T. tonsurans*	Yes	No	[77]
2013		F	84	AK/TCS	Parietal	No	+	+	-	*Trichophyton* spp.	Yes	No	[16]
2013		F	75	AK, CA/TCS, IS	Parietal, Temporal	No	-	+	+	*Microsporum* spp.	Yes	No	[16]
2013		F	93	OCS, TCS, IVCS, IS	Parietal, Temporal, Frontal	No	NA	+	+	*Microsporum* spp.	Yes	Yes	[16]
2014		F	68	TCS	Parietal	No	-	+	-	*Trichophyton* spp.	Yes	No	[78]
2016		F	79	TCS, NH	Parietal	No	NA	+	+	*T. tonsurans*	Yes	No	[79]
2016		F	72	PE	Parietal	No	NA	+	+	*Microsporum* spp.	Yes	No	[79]
2016		F	87	CA, HTN, Dement. Maln. OA, AK/NH	Parietal	No	NA	+	+	*T. rubrum*	Yes	No	[80]

TCS, topical corticosteroid; CA, cancer; PE, pet exposure; FM, family member; NH, nursing home; HS, hair salon; AK, actinic keratosis; HT, hypothyroidism; DM, diabetes mellitus; Maln., malnutrition; OCS, oral corticosteroids; HD, hair dye; Gast., gastritis; IS, immunosuppressive agent; LD, liver disease; HTN, hypertension; HIV, human immunodeficiency virus; Inf., infection; D, depression; AID, autoimmune disease; IHD, ischemic heart disease; Dement, dementia; OA, osteoarthritis.

**Table 2:** Univariate and multivariate analyses of the associations between sex, risk factors, comorbidities, etiological agent, tinea type and treatment (OR, 95% Confidence Intervals, *p*).

						Univariate Analysis			Multivariate Analysis		
Variables		Total N = 91	Cured N = 83	Not Cured N = 8	*x* ^2^	OR	CI 95%	*p*	OR	CI 95%	*p*
Sex	Female Male	82 (100%) 9 (100%)	75 (91.5%) 8 (88.9%)	7 (8.5%) 1 (11.1%)	0.06704	1.339	0.1067 to 10.42	0.7957	6.184	0.2330 to 113	0.2014
Risk Factors	Yes No	51 (100%) 40 (100%)	49 (96.1%) 34 (85%)	2 (3.9%) 6 (15%)	3.431	4.324	0.9890 to 21.73	0.0640	2.140	0.1765 to 52.05	0.5623
Comorbidity	Yes No	30 (100%) 61 (100%)	26 (86.7%) 57 (93.4%)	4 (13.3%) 4 (6.6%)	1.152	0.4561	0.1260 to 1.681	0.2832	2.712	0.2583 to 31.07	0.3840
Etiological agent	*Trichophyton* spp. Other	56 (100%) 35 (100%)	51 (91.1%) 32 (91.4%)	5 (8.9%) 3 (8.6%)	0.003426	0.9563	0.2403 to 4.029	0.9533	0.3782	0.01540 to 4.225	0.4569
Inflammatory Missing = 5	Yes No	43 (100%) 31 (100%)	39 (90.7%) 27 (87.1%)	2 (4.7%) 1 (3.2%)	0.06830	0.7222	0.04824 to 6.479	0.7938	2.032	0.1892 to 29.09	0.5566
Treatment	Yes No	85 (100%) 6 (100%)	82 (96.5%) 1 (16.7%)	3 (3.5%) 5 (83.3%)	44.51	136.7	14.88 to 1597	<0.0001 *	324.3	18.88 to 21,695	0.0008 *

* Statistically significant differences *p* ≤ 0.05.

## Data Availability

The original contributions presented in this study are included in the article. Further inquiries can be directed to the corresponding author.

## Appendix A

**Table A1:** Clinical features, comorbidities, and outcomes of elderly tinea capitis cases.

Year	Country	Misdiagnosed	Onset (Days)	Elementary Lessions (Onset)	Secondary Lessions (Evolution)	Associated Symptoms	Erythema	Alopecia	Dermoscopy	Pattern (Kerion, Favus, Dry Scalp)	Cure	Scopus Number
2007	Austria	Eczema	60	Papule	Scale	Pruritus	Yes	Yes	No	Kerion	Yes	[20]
1980	Belgium	No	60	Plaque, Pustule	Scale	Pain	Yes	Yes	No	Kerion	Yes	[21]
2014		No	NA	Pustule	Crust, Scale	Pruritus	Yes	Yes	No	Dry scalp	Yes	[22]
2014		No	NA	None	Scale	Pruritus	Yes	Yes	No	Dry scalp	Yes	[22]
2014		No	NA	Papule	Scale	Pruritus	Yes	Yes	No	Dry scalp	Yes	[22]
2014		No	NA	Papule	Scale	Pruritus	Yes	Yes	No	Dry scalp	Yes	[22]
1995	Canada	No	42	Pustule	Scale, Crust	None	Yes	Yes	No	Kerion	Yes	[23]
2000		Bacterial pyoderma	90	Pustule	Crust	None	Yes	Yes	No	Kerion	Yes	[24]
2021	Czech Republic	Seborrheic dermatitis, Psoriasis	180	Papule	Crust, Scale	Pruritus	Yes	Yes	Yes	Dry scalp	Yes	[25]
2022		No	540	Pustule	Crust	None	Yes	Yes	No	Kerion	Yes	[26]
2022		No	60	Plaque	Crust, Scale	Pain, odor	Yes	Yes	No	Favus	Yes	[27]
2023		Bacterial infection	21	Nodule, Pustule, Plaque	Crust	Pruritus, Fever, Lymphadenopathy, Pain	Yes	Yes	Yes	Kerion	Yes	[28]
2023		Seborrheic dermatitis	180	Papule	Scale, Crust	Pruritus	Yes	Yes	Yes	Dry scalp	Yes	[29]
2024		Erosive Pustular Dermatosis	90	Pustule, Nodule	Crust	Pruritus, Pain	Yes	Yes	No	Kerion	No	[30]
2025		No	180	Papule, Pustule, Patch	Ulcer, Crust	Pruritus, Fever, Pain	Yes	Yes	Yes	Kerion	Yes	[31]
2010		No	360	Pustule, Plaque	Crust	Pain	Yes	Yes	No	Kerion	Yes	[32]
2005	Germany	Bacterial pyoderma	42	Pustule, Nodule	Scale	Pruritus, Pain	Yes	Yes	No	Kerion	Yes	[33]
2011	Greece	Seborrheic dermatitis	60	Pustule	Scale, Crust	None	Yes	Yes	No	Dry scalp	Yes	[34]
2006	India	LPP	720	Papule, Macule	Scale	Pruritus	Yes	Yes	No	Kerion	Yes	[35]
2014		Bacterial pyoderma	270	Pustule, Plaque, Papule	Crust	Pruritus, Pain, Lymphadenopathy	Yes	Yes	No	Kerion	Yes	[36]
1986	Italy	No	360	Macule	Scale, Crust	Pruritus	Yes	Yes	No	Kerion	Yes	[37]
1995		No	60	Pustule, Macule	Scale	No	Yes	Yes	No	Kerion	Yes	[38]
2003		No	21	Plaque	Crust	No	Yes	Yes	No	Favus	NA	[39]
2019		No	NA	Plaque	Scale	Pruritus	Yes	Yes	Yes	Dry scalp	Yes	[40]
2022		No	>90	Papule, Pustule	Crust	Pruritus, Edema	Yes	Yes	Yes	Kerion	Yes	[41]
1998		Seborrheic dermatitis	21	Papule	Scale	Pruritus	Yes	Yes	No	Dry scalp	Yes	[42]
1999	Japan	Perifolliculitis	90	Pustule, Abscess	Scale, Crust	Pain, Pruritus	Yes	Yes	No	Kerion	Yes	[43]
2004		No	NA	None	Scale	No	No	Yes	No	Dry scalp	No	[44]
2011		No	NA	None	Scale	No	Yes	Yes	No	Dry scalp	Yes	[45]
2016		Erosive Pustul Dermatosis	60	Pustule, Plaque	Crust, Ulcer	No	Yes	Yes	No	Kerion	Yes	[46]
2021		Annular erythema	360	None	Scale	No	No	Yes	Yes	Dry scalp	Yes	[47]
2002	Mexico	No	3600	Papule	Scale, Crust	Pruritus	Yes	Yes	No	Dry scalp	Yes	[48]
2005		Discoid lupus erythematosus	375	Pustule	Scale, Crust	Pain	Yes	Yes	No	Kerion	Yes	[49]
2015		No	1040	Papule	Scale	No	Yes	Yes	Yes	Dry scalp	Yes	[50]
2024	Poland	No	24	Papule, Pustule	Crust, Scale	Pruritus	Yes	Yes	No	Kerion	Yes	[51]
2019	Portugal	Erosive Pustular Dermatosis	90	Pustule	Crust, Ulcer	Odor, Pain	Yes	Yes	No	Kerion	No	[52]
2019		No	NA	None	None	No	Yes	Yes	No	NA	Yes	[53]
2019		No	NA	None	None	No	Yes	Yes	No	NA	Yes	[53]
2019		Seborrheic dermatitis	NA	None	None	No	Yes	Yes	No	NA	Yes	[53]
2020	South Korea	No	42	Patch	Scale	Pain, Pruritus	Yes	Yes	Yes	Dry scalp	Yes	[54]
2022		Seborrheic dermatitis	1080	Patch, Pustule	Crust, Scale, Ulcer	Pruritus	Yes	Yes	No	Kerion	Yes	[55]
2024		No	180	Pustule, Patch, Papule	Crust, Scale	Pruritus	Yes	Yes	Yes	Dry scalp	Yes	[56]
2002	Spain	Erosive Pustula Dermatosis	90	Pustule, Plaque	Crust, Ulcer	Pain	Yes	Yes	No	Kerion	Yes	[57]
2002		Seborrheic dermatitis	360	Pustule, Papule	Scale	Pruritus	Yes	Yes	No	Dry scalp	Yes	[58]
2004		Cancer	1080	Papule, Pustule	Crust, Scale	No	Yes	Yes	No	Kerion	Yes	[59]
2004		No	NA	Papule, Vesicule, Pustule, Patch	Crust	Lymphadenopathy	Yes	Yes	No	Kerion	Yes	[60]
2006		No	150	None	Scale	Pruritus	Yes	Yes	No	Dry scalp	Yes	[61]
2007		No	180	Papule	Scale	Pruritus	Yes	Yes	No	Dry scalp	Yes	[62]
2012		Eczema	90	Pustule	Scale, Crust	Pruritus	Yes	Yes	No	Dry scalp	Yes	[63]
2012		Eczema	90	Pustule	Crust	Pruritus	Yes	Yes	No	Kerion	Yes	[63]
2012		No	42	None	Crust, Ulcer	No	Yes	Yes	No	Dry scalp	Yes	[63]
2012		Bacterial infection	21	None	Crust, Ulcer	Lymphadenopathy	Yes	Yes	No	Kerion	Yes	[63]
2015		Bacterial pyoderma	150	Pustule	Crust	Pruritus	Yes	Yes	No	Kerion	Yes	[64]
2016		No	NA	Pustule, Papule	Crust, Scale	No	Yes	Yes	No	NA	Yes	[5]
2016		No	NA	Papule, Pustule	Scale, Crust	No	Yes	Yes	No	NA	Yes	[5]
2016		No	NA	Papule, Pustule	Scale, Crust	No	Yes	Yes	No	NA	Yes	[5]
2016		No	NA	Papule, Pustule	Scale, Crust	No	Yes	Yes	No	NA	Yes	[5]
2016		No	NA	Papule, Pustule	Scale, Crust	No	Yes	Yes	No	NA	Yes	[5]
2016		No	NA	Papule, Pustule	Scale, Crust	No	Yes	Yes	No	NA	Yes	[5]
2016		No	NA	Papule, Pustule	Scale, Crust	No	Yes	Yes	No	NA	Yes	[5]
2016		No	NA	Papule, Pustule	Scale, Crust	No	Yes	Yes	No	NA	Yes	[5]
2016		No	NA	Papule, Pustule	Scale, Crust	No	Yes	Yes	No	NA	Yes	[5]
2016		No	NA	Papule, Pustule	Scale, Crust	No	Yes	Yes	No	NA	Yes	[5]
2016		No	NA	Papule, Pustule	Scale, Crust	No	Yes	Yes	No	NA	Yes	[5]
2016		No	NA	Papule, Pustule	Scale, Crust	No	Yes	Yes	No	NA	Yes	[5]
2016		No	NA	Papule, Pustule	Scale, Crust	No	Yes	Yes	No	NA	Yes	[5]
2016		No	NA	Papule, Pustule	Scale, Crust	No	Yes	Yes	No	NA	Yes	[5]
2021		No	90	Pustule	Crust, Ulcer	No	Yes	Yes	No	Kerion	Yes	[65]
2021		No	365	Plaque	Scale	No	Yes	Yes	Yes	Dry scalp	Yes	[66]
1991	Taiwan	Seborrheic dermatitis	7200	Pustule, None	Scale	Pruritus	Yes	Yes	No	Dry scalp	Yes	[67]
1991		No	30	Pustule, Papule	Scale, Crust	Pruritus	Yes	Yes	No	Kerion	Yes	[67]
1991		No	30	Pustule, Papule	Scale, Crust	Pruritus	Yes	Yes	No	Dry scalp	Yes	[67]
2014		No	30	Papule, Macule	None	Pain	Yes	Yes	Yes	Dry scalp	NA	[68]
2015		No	90	Pustule, Plaque	None	No	Yes	Yes	Yes	Dry scalp	NA	[69]
2012	Tunisia	No	3600	Plaque	Scale, Crust	Pain	Yes	Yes	No	Favus	Yes	[70]
1978	United Kingdom	No	360	Papule	Scale, Crust	No	Yes	Yes	No	Kerion	Yes	[71]
1994		Erosive Pustular Dermatosis	28	Pustule	Scale, Crust	No	Yes	Yes	No	Kerion	Yes	[72]
2001		No	90	Pustule	Scale	No	Yes	Yes	No	Dry scalp	Yes	[73]
2001		No	120	Pustule, Papule	Scale	Edema	Yes	Yes	No	Kerion	Yes	[73]
1980	USA	No	10	Pustule, Plaque	Crust, Scale	Pain	Yes	Yes	No	Kerion	Yes	[74]
1993		Discoid lupus erythematosus	NA	Plaque, Pustule	Scale	Pruritus	Yes	Yes	No	Dry scalp	NA	[75]
2002		Seborrheic dermatitis	1800	None	Scale	Pruritus	Yes	Yes	No	Dry scalp	NA	[76]
2003		Bacterial pyoderma	720	Pustule	Scale, Crust	Pruritus, Pain, Lymphadenopathy	Yes	Yes	No	Kerion	Yes	[77]
2003		Bacterial pyoderma	15	Pustule	Crust	Lymphadenopathy, Pain	Yes	Yes	No	Kerion	Yes	[77]
2013		Erosive Pustular Dermatosis	90	Pustule	Scale, Ulcer, Crust	Pain	Yes	Yes	No	Kerion	Yes	[16]
2013		Erosive Pustular Dermatosis	720	Pustule, Nodule	Scale, Ulcer, Crust	Pain	Yes	Yes	No	Kerion	Yes	[16]
2013		Erosive Pustular Dermatosis	1080	Pustule, Nodule	Ulcer, Crust	Pain	Yes	Yes	No	Kerion	Yes	[16]
2014		No	90	Pustule, Nodule	None	Pruritus, Pain	Yes	Yes	Yes	Kerion	Yes	[78]
2016		No	180	Patch	Scale	Pruritus	Yes	Yes	Yes	Dry scalp	Yes	[79]
2016		No	720	Patch	Scale	Pruritus	Yes	Yes	No	Dry scalp	Yes	[79]
2016		No	>90	Plaque, Nodule	NA	No	Yes	Yes	No	Favus	Yes	[80]
